# *Trichoderma* spp., *Bacillus* spp. and P*seudomonas* spp. isolated from commercial products show biocontrol activity against soil-borne pathogens in soybean

**DOI:** 10.1038/s41598-026-67813-2

**Published:** 2026-08-28

**Authors:** Franziska Porsche, Marwin S. Nagel, Anja Frank, Andreas Hammelehle, Ada Linkies

**Affiliations:** 1 Julius Kühn-Institut (JKI), Bundesforschungsinstitut für Kulturpflanzen, Institut für Biologischen Pflanzenschutz, Schwabenheimer Str. 101, 69221 Dossenheim, Germany; 2 Fachinformation Ökologischer Landbau, Landesbetrieb Landwirtschaft Hessen, Beratungsstelle Friedberg, Homburger Straße 17, 61169 Friedberg, Germany

**Keywords:** Biological control, Seed treatment, Rhizoctonia solani, Diaporthe longicolla, Sclerotinia sclerotiorum, Fusarium oxysporum, Volatile organic compound (VOC), Glycine max, Biotechnology, Microbiology, Plant sciences

## Abstract

This study evaluated the biocontrol potential of ten commercially available microbial soil additives and fungicides against major soil-borne pathogens (*Rhizoctonia solani*, *Fusarium oxysporum*, *Sclerotinia sclerotiorum* and *Diaporthe longicolla*) to identify immediate, market-ready solutions for farmers, in the case of soil additives without the delay of product registration. In vitro dual cultures demonstrated strain- and pathogen-dependent antagonistic activity, with *Pseudomonas* and *Bacillus* being less effective than the tested *Trichoderma* strains. Regarding bacterial antagonists, the production of volatile organic compounds (VOCs) and lytic enzymes was confirmed in vitro, indicating potential mechanisms of antagonism. Compatibility tests on soybean plants demonstrated no negative effects on germination, overall growth, or nodule abundance in nearly all cases. In greenhouse bioassays, under high *R. solani* pressure (1.5%) microbial treatments did not significantly restore germination rate and seedling growth. However, *T. harzianum* T50 (Vitalin Trichoderma) and *T. harzianum* + *P. fluorescens* mixture (Trillus), significantly reduced hypocotyl lesion severity and increased the proportion of symptom-free seedlings. Overall, *in planta* efficacy was highly variable compared to the strong in vitro inhibition. Nevertheless, selected commercial strains show clear potential to mitigate severe disease symptoms under high *R. solani* pressure.

**Supplementary Information:**

The online version contains supplementary material available at https://doi.org/10.1038/s41598-026-67813-2.

## Introduction

Soybean (*Glycine max* L. Merr.) is a leguminous crop of major global importance, serving as one of the main protein sources for animal production and playing a key role both in sustainable cropping systems and as a food source in the human diet^1,2^. Although the United States and Brazil lead global production^3,4^, European soybean cultivation is steadily increasing^5^, driven by the demand for locally produced, non-genetically modified soy^6^, the breeding of adapted cultivars, and the increasing temperatures in Central and Northern Europe due to climate change. In addition, there is a trend toward lowering the demand for meat products, while increasing the need for plant-based food alternatives, among which soy plays a significant role^7^. This aligns with European efforts to promote protein crops, which in Germany is implemented as part of the “Protein Crop Strategy”^8^. Subsequently, soybean cultivation in Germany has expanded in recent years; however, abiotic stress, such as weather extremes and prolonged periods of low temperatures, influence crop success, overall yield, and yield stability^9,10^.

Infections by soil-borne pathogens significantly reduce stand establishment and yield under European growing conditions. Pathogens such as *Fusarium* spp., *Sclerotinia sclerotiorum*, *Rhizoctonia solani*, and *Diaporthe* species are among the most economically important fungal pathogens affecting soybean production worldwide^11,12^. They infect soybean plants at different developmental stages and colonize seeds, roots, stems, and pods, causing diseases such as seed decay, damping-off, root rot, stem canker, pod and stem blight, and white mold. They reduce plant vigor, ultimately resulting in reduced seed quality and yield. Among these pathogens, *Sclerotinia sclerotiorum* is one of the most destructive, with reported soybean yield losses of up to 50% during severe epidemics^11,13^. Management of these pathogens can be achieved through cultivar selection, seed dressing, and the application of chemical pesticides. In both organic and integrated systems, reducing the use of chemical-synthetic plant protection products is a central objective aligned with the German “2035 Arable Farming Strategy” and the EU-based “Farm to Fork Strategy”, promoting sustainable aspects in agriculture. Furthermore, the use of chemical-synthetic agents is prohibited in organic production, which requires non-chemical alternatives to ensure crop success.

Microbial antagonists, including beneficial *Trichoderma*, *Bacillus*, and *Pseudomonas* species, represent a promising tool for both integrated disease management and plant-growth promotion^14–16^. Beneficial bacteria and fungi suppress pathogens through mechanisms such as niche competition, antibiosis, mycoparasitism, and the induction of induced resistance^17,18^. Although these genera are widely described as effective suppressors of soil-borne pathogens in major crops like maize and wheat^16–21^, their systematic evaluation in emerging European soybean systems remains limited. Recent research emphasizes that the practical success of microbial biocontrol relies heavily on understanding specific host-rhizosphere interactions and overcoming the variable efficacy often observed between lab and field conditions^14,22^.

A unique challenge in soybean cultivation is ensuring that these broad-spectrum microbial antagonists do not interfere with *Bradyrhizobium japonicum.* These root-nodule bacteria form a symbiosis with soybean roots and enable biological nitrogen fixation. However, these bacteria are not naturally present in Central European soils and must be co-inoculated during sowing to guarantee stable yields^23–25^.

To date, a significant research gap exists regarding how established, commercially available biocontrol strains interact within this tripartite system (host plant – pathogens – symbionts) in soybean. This study aimed to address this gap by evaluating the potential of ten commercial microbial products to promote soybean plant growth while simultaneously providing protection against the soil-borne pathogens *R. solani*, *F. oxysporum*, *S. sclerotiorum* and *D. longicolla*, and ensuring compatibility with *B. japonicum*. To avoid long phases of product development and registration procedures from the beginning, we strategically focused on commercially available microbial products already approved in Germany for other crops or registered as growth promoters for general use. The tested products contain species of the genera *Trichoderma*, *Pseudomonas*, and *Bacillus*. The antagonistic potential of the microorganisms against the target fungi was evaluated in vitro using dual-culture assays. Furthermore, we investigated whether the microorganisms showed defense-related enzyme activity or volatile organic compound (VOC) production and activity. The most suitable candidates based on our in vitro trials were subsequently tested in soybean plant trials upon soil inoculation with *R. solani*. Seed furrow treatments were prioritized, as this step can be easily combined with the sowing of rhizobia-inoculated seeds, as is common practice. Consequently, the results obtained here are meant to be readily transferable into soybean cultivation systems in Germany.

## Results

### Effect of bacterial and fungal antagonists on pathogen growth in dual culture assays

Dual culture assays were used to investigate the ability of five bacterial and five fungal strains, previously isolated from microbial products (Table 1), to inhibit the mycelial growth of the soilborne pathogens *R. solani*, *F. oxysporum*, *S. sclerotiorum* and *D. longicolla*.

**Table 1:** Microbial strains and products used in this study. The microbial strains were isolated from the respective products as indicated. n.s.: strain name not specified.

Organism	^1^Abbreviation	Product name	Manufacturer	*Product dosage (in water)
*Bacillus atrophaeus* n.s.	*B. at.*	Rhizo Plus	Andermatt Biogarten AG	^2^ not applicable
*Bacillus amyloliquefaciens* (QST713)	*B. am.*	Serenade Aso	Bayer AG	160 µl/100 ml
*Bacillus velezensis* (FZB42)	*B. ve.*	Rhizo Vital	Biofa GmbH	40 µl/100 ml
*Pseudomonas fluorescens* (B177-M-03.08) ^3^ *Trichoderma harzianum* (B97-M-04.08)	*P. fl.* *T. har.*	Trillus	Agroplanta GmbH & Co. KG	110 mg/100 ml
*Pseudomonas chlororaphis* (MA342)	*P. chl.*	Cerall	Intrachem Bio Deutschland GmbH & Co. KG	^2^ not applicable
^3^*Pseudomonas* spp. n.s. ^3^*Glomus* spp. n.s. *Trichoderma* spp. n.s.	*P.* spp. *Glomus* spp. *T.* spp.	Vici Rhyzoteam GR	Koppert Deutschland GmbH	75 mg/100 ml
*Trichoderma asperellum* (Kd)	*T. asp.*	T-Gro	Biofa GmbH	50 mg/100 ml
*Trichoderma harzianum* (T50)	*T. har.* (T50)	Vitalin Trichoderma	Vitalin Pflanzgesundheit GmbH	200 mg/100 ml
*Trichoderma harzianum* (T58)	*T. har.* (T58)	Trichostar	Intrachem Bio Deutschland GmbH & Co. KG	^2^ not applicable
^4^*Trichoderma harzianum* n.s. ^4^*Trichoderma koningii* n.s.	*T. har.* *T. kon.*	Promot Plus	Intrachem Bio Deutschland GmbH & Co. KG	^2^ not applicable

^1^ Abbreviation used throughout the manuscript.

^2^ Products were not included in plant trials with *R. solani* (Fig. 7) based on poor in vitro performance of their isolated strains.

^3^ Strains are part of the commercial product, but were not used in in vitro trials in this study due to experimental reasons.

^4^ Strains were used jointly in this study, separation was not possible.

* Product dosage and application quantity as used for soil treatment in plant trials.

**Fig. 1:**
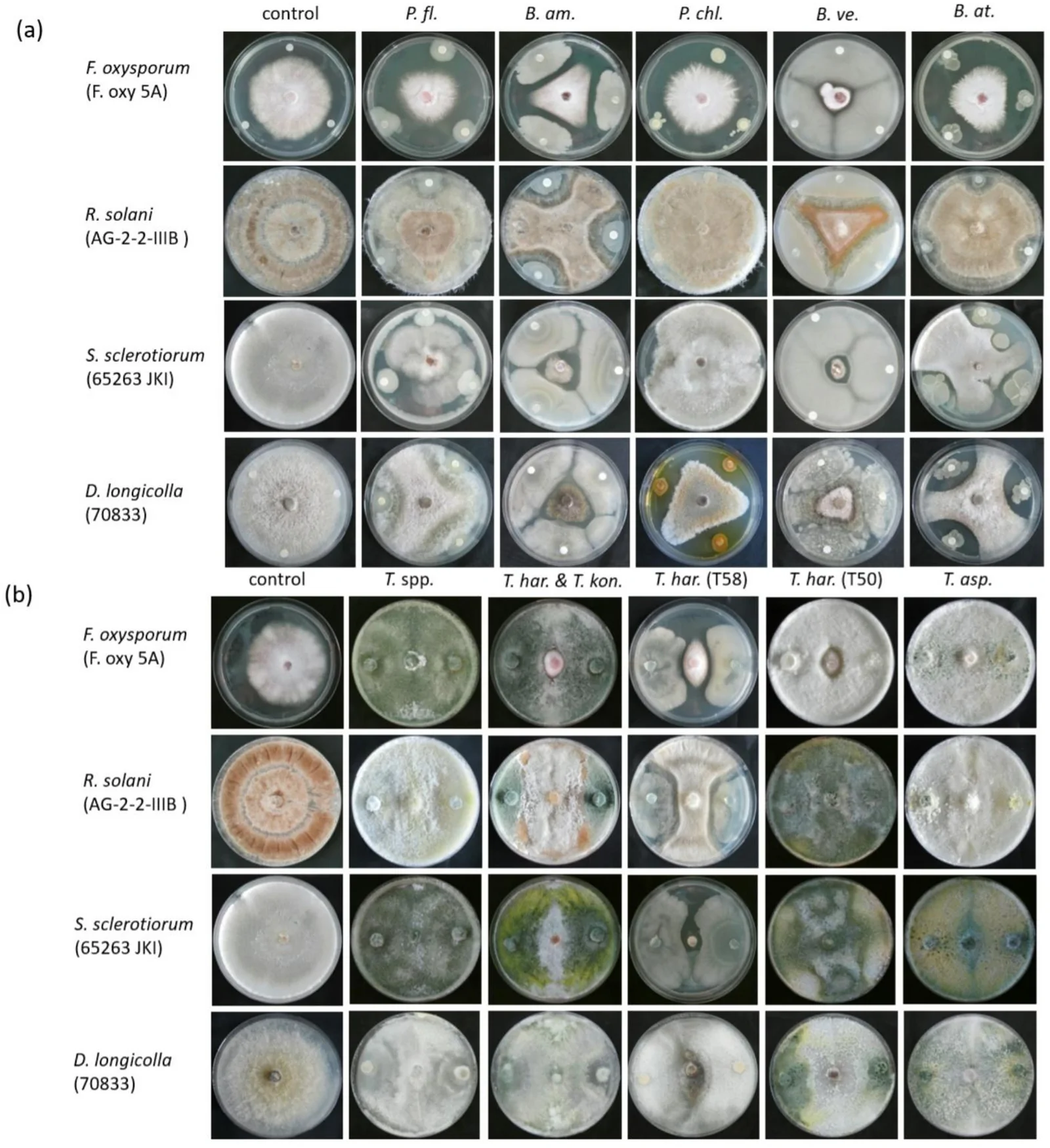
Suppressive effects of bacterial (**a**) and fungal antagonists (**b**) on mycelial growth of *Fusarium oxysporum* (F. oxy 5 A), *Rhizoctonia solani* (AG-2-2-IIIB), *Sclerotinia sclerotiorum* (65263 JKI), and *Diaporthe longicolla* (70833) determined in dual culture plate assays. Mycelial disks of the pathogens were placed in the middle of a Petri dish on potato dextrose agar (PDA), while the antagonists were placed at three (bacteria) or two (*Trichoderma* spp.) locations next to the pathogens. Images were taken after four days incubation at 26 °C. The growth inhibition rates (%) are shown in Fig. 2. *B. am*.: *Bacillus amyloliquefaciens* (QST713); *B. at*.: *Bacillus atrophaeus* n.s.; *B. ve*.: *Bacillus velezensis* (FZB42); *P. chl*.: *Pseudomonas chlororaphis* (MA342); *P. fl.*.: *Pseudomonas fluorescens* (B177-M-03.08); *T. asp*.: *Trichoderma asperellum* (Kd); *T. har*. & *T. kon*.: *Trichoderma harzianum* n.s. & *Trichoderma koningii* n.s.; *T. har*. (T58): *Trichoderma harzianum* (T58); *T. har*. (T50): *Trichoderma harzianum* (T50); *T*. spp.: *Trichoderma* spp. n.s.; control: treatment without antagonist. *n* = 9. Representative pictures of each treatment are shown.

**Fig. 2:**
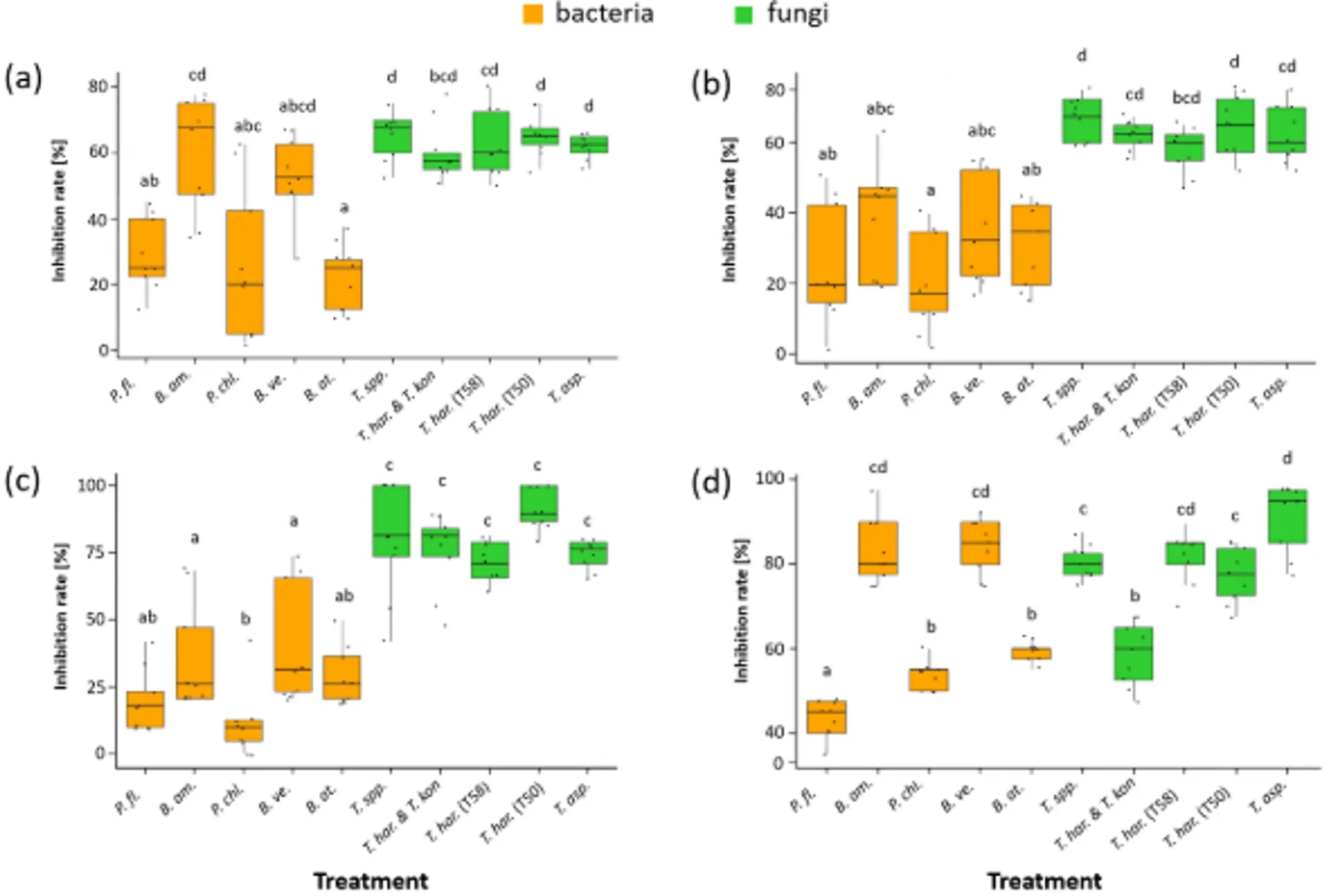
Mycelial growth inhibition (%) of bacterial (orange) and *Trichoderma* (green) antagonists on soil-borne pathogens determined in dual culture plate assays. Mycelial disks of the pathogens were placed in the middle of a Petri dish on PDA, while the antagonists were placed at three (bacteria) or two (*Trichoderma* spp.) locations next to the pathogen. Growth inhibition rates (%) against (**a**) *Rhizoctonia solani* (AG-2-2-IIIB), (**b**) *Fusarium oxysporum* (F. oxy 5 A), (**c**) *Sclerotinia sclerotiorum* (65263) and (**d**) *Diaporthe longicolla* (70833) were determined after 4 to 6 days relative to the nontreated control. *B. am*.: *Bacillus amyloliquefaciens* (QST713); *B. at*.: *Bacillus atrophaeus* n.s.; *B. ve*.: *Bacillus velezensis* (FZB42); *P. chl*.: *Pseudomonas chlororaphis* (MA342); *P. fl.*.: *Pseudomonas fluorescens* (B177-M-03.08); *T. asp*.: *Trichoderma asperellum* Kd; *T. har*. & *T. kon*.: *Trichoderma harzianum* n.s. & *Trichoderma koningii* n.s.; *T. har.* (T58): *Trichoderma harzianum* (T58); *T. har*. (T50) *Trichoderma harzianum* (T50); *T*. spp.: *Trichoderma* spp. n.s.; The bars show the mean ± standard deviation. *n* = 9. The bars and circles indicate medians and data points, respectively. Boxes indicate the interquartile range (25th–75th percentile) and whiskers indicate lower and upper quartiles. Letters above the treatments denote statistically significant differences with *p* < 0.05 according to Dunn´s test.

The bacterial strains exhibited different levels of antagonistic activity. *Bacillus amyloliquefaciens* and *Bacillus velezensis* inhibited the fungal growth of *R. solani* by 59% and 53%, respectively (Figs. 1a and 2a), whereas the other bacterial strains (*Pseudomonas fluorescens*, *Pseudomonas chlororaphis*, *Bacillus atrophaeus*) showed comparatively lower inhibitory effects, ranging from 23% to 29%. This separation was statistically supported by the Dunn´s test, which placed *B. am.* in higher significance groups compared to the other bacterial strains.

In dual culture assays with *F. oxysporum* and *S. sclerotiorum*, all bacterial antagonists resulted in lower levels of growth inhibition (Fig. 2b, c), with a maximum of 40% for *B. velezensis*. The inhibitory activity of the bacterial strains against *D. longicolla* was higher (Fig. 2d). *B. amyloliquefaciens* and *B. velezensis* showed the highest activity, reducing the mycelial growth of *D. longicolla* by more than 83%, whereas the other bacterial strains had a significantly lower effect (Dunn´s test, *p* < 0.05), leading to inhibition rates of 43% − 59%. The relative inhibitory effect when comparing the bacterial strains with each other showed a similar relative activity for all pathogens.

In contrast, all *Trichoderma* strains demonstrated consistently stronger antifungal activity, reducing mycelial growth of all four pathogens by at least 59% (Figs. 1 and 2). For *R. solani* and *F. oxysporum*, the fungal isolates clustered in the highest significance groups, significantly outperforming most bacterial treatments (*p* < 0.05). Against *S. sclerotiorum*, all *Trichoderma* strains demonstrated a highly homogeneous and strong inhibitory effect, sharing the identical significance group. No significant differences in the reduction of relative fungal growth were observed among the *Trichoderma* strains, except against *D. longicolla*. Here, the combination of *T. harzianum* and *T. koningii* (Promot Plus) was significantly less effective (Dunn´s test, *p* < 0.05), whereas *T. asperellum* achieved the highest efficacy, being statistically comparable only to *T. harzianum* (T58). Overall, these results highlight *Trichoderma* species as most potent antagonists across all tested pathogens. In comparison, bacterial species showed lower efficacy, though *Bacillus* strains consistently demonstrated higher activity compared to the *Pseudomonas* strains.

### Effect of bacterial volatile organic compounds on fungal pathogen growth

**Fig. 3:**
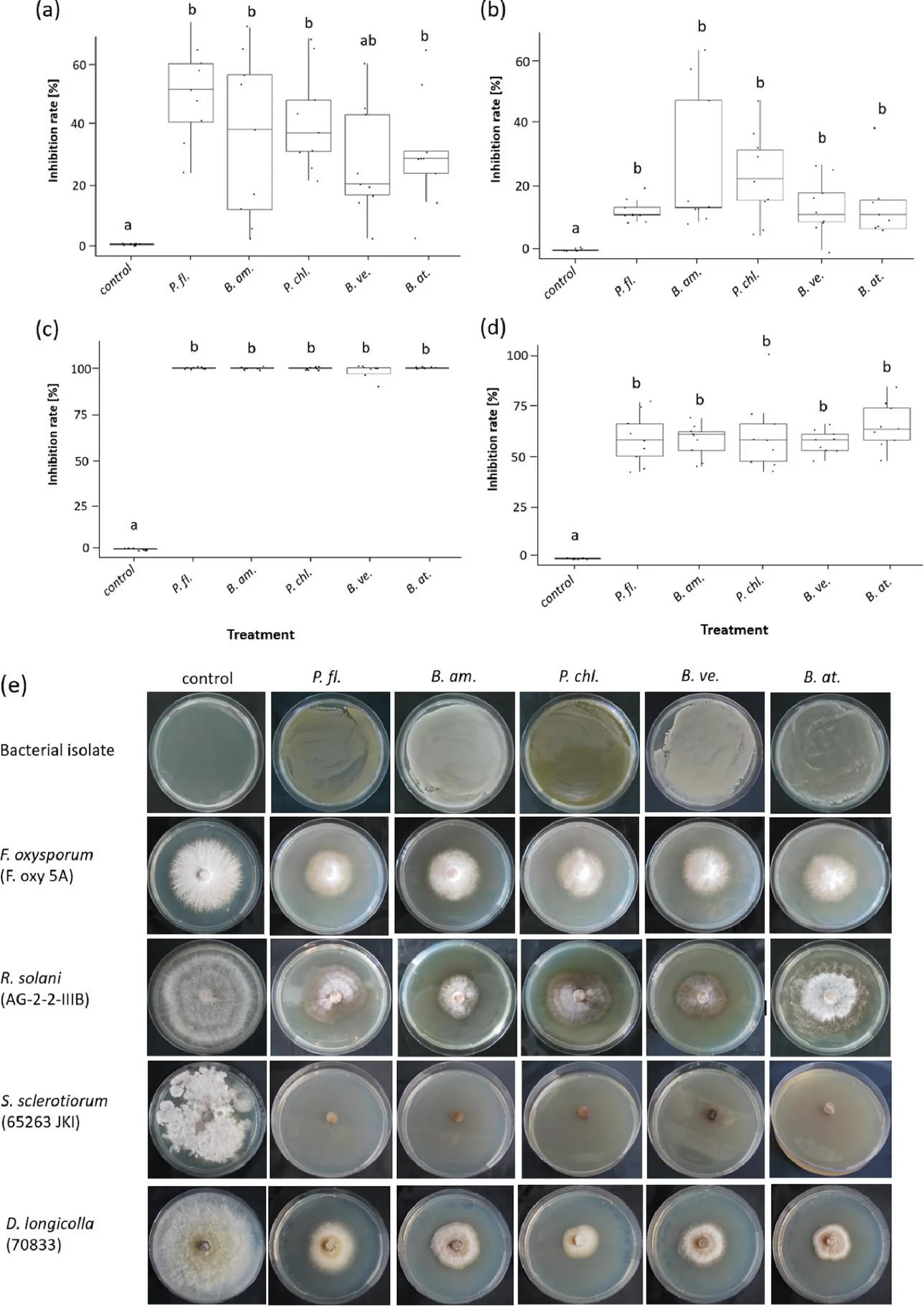
Growth inhibition (%) of bacterial volatile organic compounds (VOCs) on fungal pathogens in double-chamber assays. (**a**) *Rhizoctonia solani* (AG-2-2-IIIB), (**b**) *Fusarium oxysporum* (F. oxy 5 A), (**c**) *Sclerotinia sclerotiorum* (65263 JKI), and (**d**) *Diaporthe longicolla* (70833) were applied simultaneously with bacterial and fungal antagonists on opposite plates of Petri dishes and sealed with parafilm. (**e)** Representative pictures of each treatment after three days incubation are shown. *B. am*.: *Bacillus amyloliquefaciens* (QST713); *B. at*.: *Bacillus atrophaeus* n.s.; *B. ve*.: *Bacillus velezensis* (FZB42); *P. chl*.: *Pseudomonas chlororaphis* (MA342); *P. fl.*.: *Pseudomonas fluorescens* (B177-M-03.08); control: treatment without antagonist. Inhibition rates were determined after three days incubation at 26 °C and calculated in comparison to the untreated control. The bars and circles indicate medians and data points, respectively. Boxes indicate the interquartile range (25th–75th percentile) and whiskers indicate lower and upper quartiles. Letters above the treatments denote statistically significant differences with *p* < 0.05 according to Dunn´s test, *n* = 9.

The effect of bacterial volatile organic compounds (VOCs) on the mycelial growth of *R. solani*, *F. oxysporum*, *S. sclerotiorum* and *D. longicolla* was tested using the double-plate chamber method, where two plates are fixed together with parafilm, each of them containing one of the individual organisms. This allows analysis of VOC-mediated influences on pathogen growth. The five bacterial strains isolated from the microbial products (Table 1) showed significant inhibitory effects (Dunn’s post hoc test, *p* < 0.05) against the soilborne pathogens compared to the nontreated control (Fig. 3).

The highest sensitivity to bacterial VOCs was obvious for *S. sclerotiorum*, all bacterial strains caused nearly complete inhibition of mycelial growth (98.6% − 100%). High inhibitory activity was also observed against *D. longicolla*, with VOCs from all strains reducing fungal growth by 57.0% to 66.4%. For *R. solani* and *F. oxysporum*, inhibition rates varied the most between the antagonists, but not significantly. For *R. solani*, VOCs of *B. velezensis* induced an inhibition of 27.5%, which was not significantly different from the control, while VOCs of *B. atrophaeus* caused a significant inhibition of 30.9%. VOCs of the other bacterial antagonists (*B. amyloliquefaciens*, *P. chlororaphis* and *P. fluorescens*) led to higher inhibition between 36.2% and 51.2%. VOCs of *B. amyloliquefaciens* showed the highest activity against *F. oxysporum*, reducing mycelial growth by 27.0%, while VOCs of the other strains caused minor growth reductions (12.6% − 23.5%).

Overall, the VOC-mediated inhibition was apparent for the bacterial strains against all tested fungi, indicating broad-spectrum activity of the generated VOCs. Additionally, a consistent shift in pH from + 1.75 to + 2 was recorded in all double-plate systems containing bacterial antagonists, compared to the untreated control (pH 5.5–5.75) (Table S1). When the pH value of the growth medium was artificially increased by 2 units (to pH 7.5) and fungi were placed on it without VOC treatment, growth inhibition of *D. longicolla* was observed by 32.3%. All other pathogens were not affected by exclusively changing the pH, confirming that VOC are responsible for the growth inhibition.

### Influence of bacterial volatile compounds (VOCs) on phenotypic traits

**Fig. 4:**
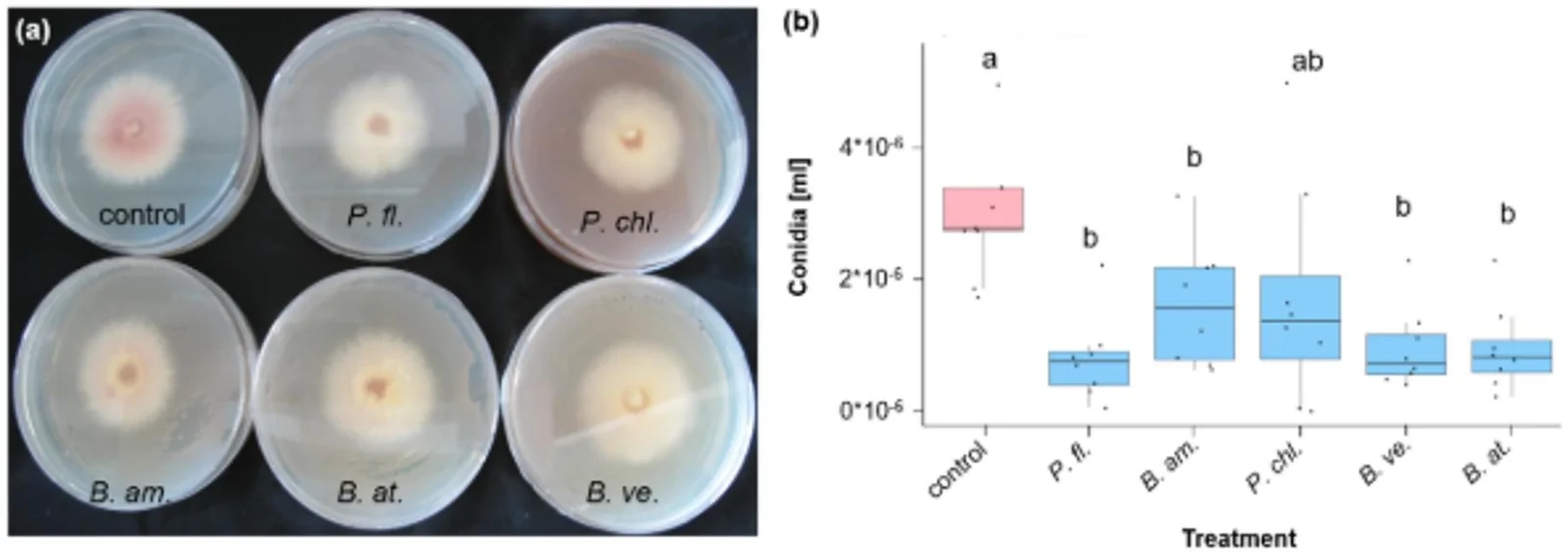
Influence of bacterial volatile organic compounds (VOCs) on morphology of *F. oxysporum* (F. oxy 5 A) analyzed in double-chamber assays. (**a**) Mycelial coloration under respective treatments, (**b**) number of conidia (conidia/mL) in response to bacterial VOC treatment. Conidia were collected by floating the grown PDA medium with 1 mL demineralized water. The bars and circles indicate medians and data points, respectively. Boxes indicate the interquartile range (25th–75th percentile) and whiskers indicate lower and upper quartiles. *n* = 9; the letters above the treatments indicate significant differences (Tukey’s test, *p* < 0.05), (*B. am*.: *Bacillus amyloliquefaciens* (QST713); *B. at*.: *Bacillus atrophaeus* n.s.; *B. ve*.: *Bacillus velezensis* (FZB42); *P. chl*.: *Pseudomonas chlororaphis* (MA342); *P. fl.*.: *Pseudomonas fluorescens* (B177-M-03.08).

VOC exposure from all bacterial strains induced phenotypic changes in the morphology of *F. oxysporum*, visible as a change in mycelium color on the underside of the Petri dish from light pink to light yellow or cream (Fig. 4a). Further, all treatments except for *P. chlororaphis* significantly reduced the number of conidia of *F. oxysporum* (Tukey’s test, *p* < 0.05) (Fig. 4b). In contrast, *R. solani*, *S. sclerotiorum*, and *D. longicolla* showed no visible changes in mycelial color or spore abundance, where applicable (not all examined species produce spores).

### Evaluation of extracellular lytic enzyme activity

**Fig. 5:**
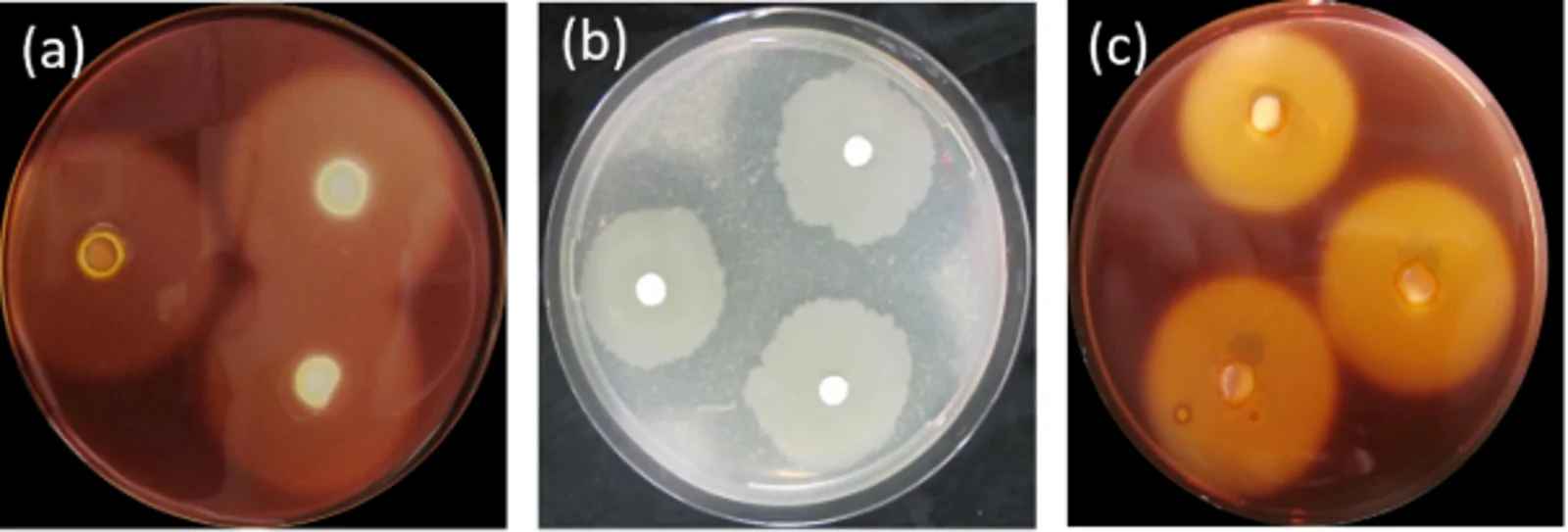
Examples of activity of extracellular hydrolytic enzymes produced by bacterial strains. The clear zones around the filter paper discs with bacterial colonies indicates enzyme activity, exemplified for (**a**) chitinase activity of *Bacillus amyloliquefaciens* QST713 on a chitinase medium (**b**) protease activity of *Pseudomonas fluorescens* B177-M-03.08 on skimmed milk agar (SM), and (**c**) cellulase activity of *Bacillus amyloliquefaciens* QST713 on carboxymethylcellulose agar (CMC). The bacterial antagonists were grown for 24 h at 26 °C and 180 rpm in TSB, cultures were adjusted to an optical density of OD₆₀₀ = 0.4 and 15 µl were pipetted onto three sterile filter paper discs (⌀ 6 mm) on each agar plate. The plates were incubated for 72 h at 26 °C in the dark. After incubation, the plates for chitinase and cellulase activity were overlaid with Gram’s iodine-potassium iodide solution for 15 min to visualize enzymatically degraded substrate.

To get insight into the mode of action of the bacterial antagonists, enzyme plate-assays were performed with the bacterial strains. All three *Bacillus* strains exhibited protease, chitinase and cellulase activity, as shown by the presence of halo formation (Fig. 5; Table 2). For *P. fluorescens*, only protease activity was evident, but no cellulase and chitinase activity. No enzyme activity was observed for *P. chlororaphis*.

**Table 2:** Analysis of extracellular hydrolytic enzyme activity of bacterial strains. Enzyme activity was based on the presence (+) or absence (-) of clearing on the respective specific substrate containing media. *n* = 9.

Organism	Chitinase	Cellulase	Protease
*P. chlororaphis* MA342	-	-	-
*P. fluorescens* B177-M-03.08	-	-	+
*B. amyloliquefaciens* QST713	+	+	+
*B. velezensis* FZB42	+	+	+
*B. atrophaeus* n.s.	+	+	+

### Influence of microbial isolates on *Bradyrhizobium japonicum* and soybean plants

The compatibility of each bacterial and fungal strain with *Bradyrhizobium japonicum* and soybean cultivar `Lenka´ was tested. In dual cultures on tryptic soy agar (TSA), no negative effect of the antagonists on *B. japonicum* growth was observed. In addition, the treatments with the microbial antagonists had no effect on seed germination rates (ANOVA, *p* > 0.05; Figure S1). Most microbial strains had no significant effect on plant height (ANOVA, *p* > 0.05 or root nodule formation (Dunn´s test, *p* > 0.05). Only *P. chlororaphis* significantly reduced plant height (ANOVA; *p* < 0.05) and led to a reduction in the number of root nodules per plant (Dunn´s test, *p* < 0.05) after 35 days.

### Development of an inoculation bioassay for *R. solani*

**Fig. 6:**
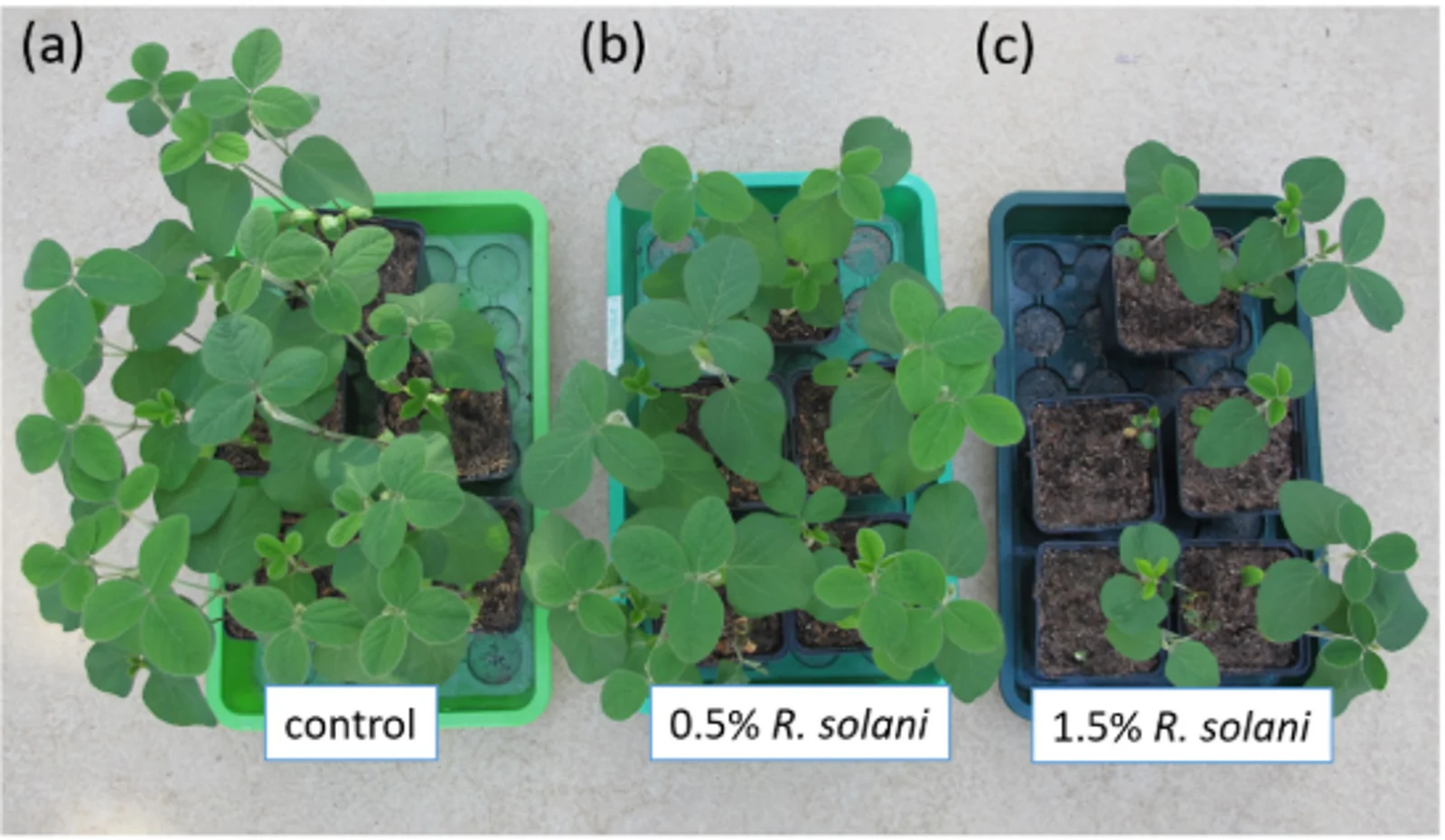
Effect of *Rhizoctonia solani* on the development of soybean plants, cv. `Lenka´ in the greenhouse. (**a**) untreated healthy control, (**b**) substrate inoculated with 0.5% *R. solani* (mL·g^− 1^ soil), and (**c**) substrate inoculated with 1.5% *R. solani* (mL·g^− 1^ soil). The pots were incubated in the greenhouse at 20–25 °C and 50% to 65% relative humidity. Lighting was adjusted so that the plants received at least 5 klux of light per day. Representative plants of each treatment are shown.

After confirming that the microorganisms did not negatively affect the growth of *B. japonicum* or seed germination rate, an inoculation bioassay for the defined infection of soybean plants with all fungal pathogens investigated here was attempted, but only for *R. solani* successfully developed. Adding *R. solani* to the soil significantly reduced germination rates and plant height in a concentration-dependent manner (Fig. 6; Table 3). Moreover, inoculating the soil with *R. solani* resulted in dose-dependent development of disease symptoms on the hypocotyl of soybean plants. A notable decrease in total plant fresh weight was also observable, although this was not significant (Table 4).

**Table 3:** Effect of *Rhizoctonia solani* on growth parameters and disease severity of soybean plants. *R. solani* (0.5%, 1.5%; mL·g^− 1^ soil) was mixed in the potting substrate of soybean seedlings cv. `Lenka´. Germination rate and plant height were determined 21 days after sowing, total fresh weight of the above-ground parts after 35 days, and disease symptoms on soybean hypocotyl, rated on a scale from 0 to 4. Disease scale: 0 = no symptoms, 1 = scattered brownish discoloration, 2 = several slightly brownish spots or lesions, 3 = numerous distinct brown or black lesions, 4 = strong or extensive black lesions (Fig. 9). Three replicates were done, each with 30 seeds per treatment. Different letters above the error bars indicate significant differences as per Tukey´s HSD test (*p* < 0.05) for germination rate and total fresh weight or Dunn´s test for plant height and disease symptom class. *n* = 90.

Treatment	Germination rate (%)	Plant height (cm)	Total fresh weight (g)	Disease symptom class
Water control	93.3 ± 6.7^a^	25.9 ± 7.8^a^	54.5 ± 19.5^a^	0.0 ± 0.0^a^
0.5% *R. solani*	57.5 ± 21.3^b^	15.5 ± 8.1^b^	27.9 ± 10.6^a^	1.7 ± 0.6^b^
1.5% *R. solani*	45.3 ± 16.3^b^	14.0 ± 8.9^b^	25.8 ± 13.5^a^	2.2 ± 0.5^b^

### Antagonistic effects of microbial antagonists on *R. solani* on soybean plants

**Fig. 7:**
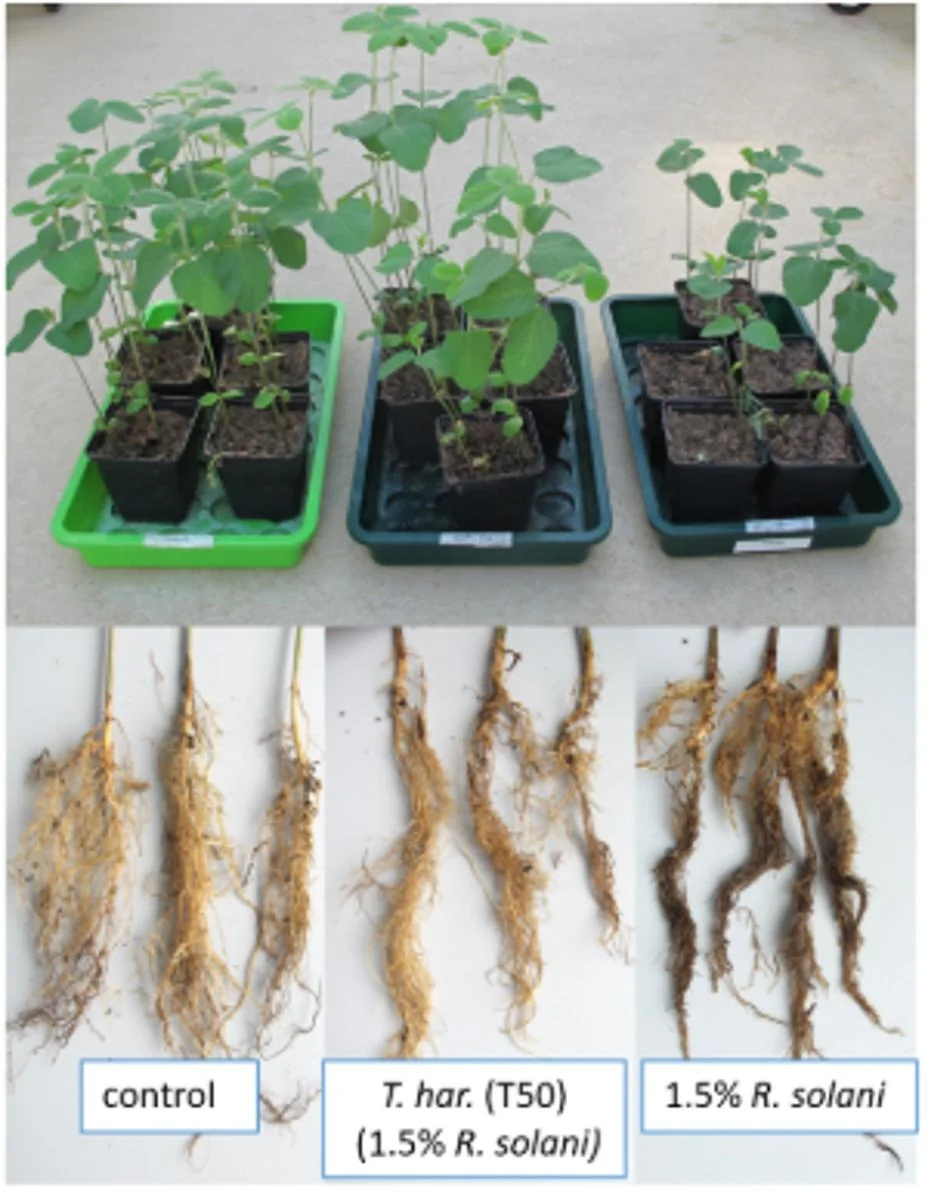
Effect of seed furrow-treatment with *T. harzianum* (T50) (Vitalin Trichoderma) on plant development of soybean cv. `Lenka´ in the greenhouse in *Rhizoctonia solani* (AG-2-2-IIIB) inoculated soil. Overview on a plant trial 35 d after inoculation. Treatments are from left to the right: untreated healthy control, seed furrow treatment with *T. harzianum* (T50) (Vitalin Trichoderma) in 1.5% *R. solani* inoculated soil and 1.5% *R. solani* inoculated treatment only. Three replicates were performed, each with 30 seeds per treatment. The pots were incubated in the greenhouse at 20–25 °C and 50–65% relative humidity. Lighting was adjusted so that the plants received at least 5 klux of light per day.

**Fig. 8:**
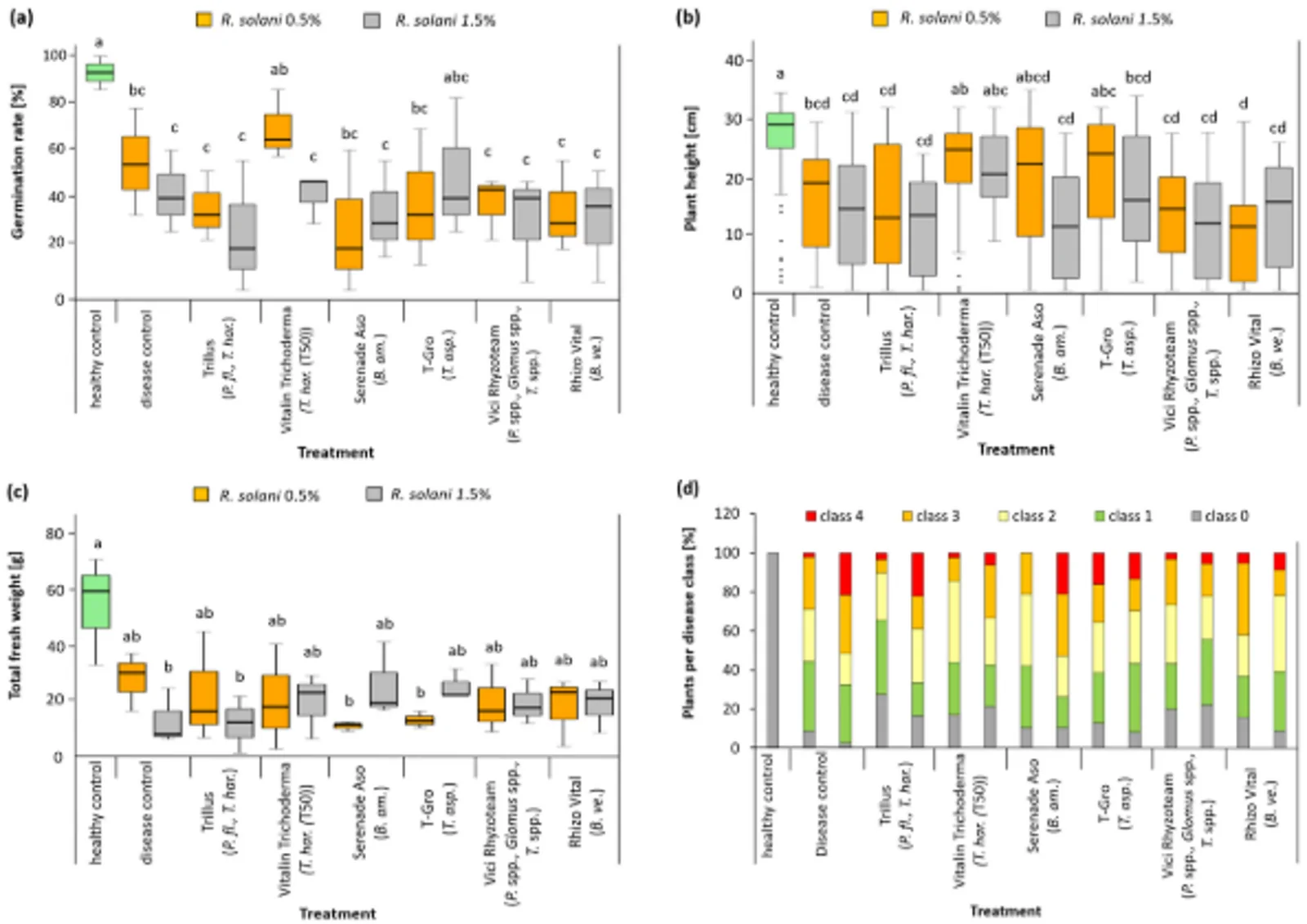
Effect of microbial biocontrol products on disease severity of *Rhizoctonia solani* (AG-2-2-IIIB) and related plant parameters of soybean plants cv. `Lenka´. Influence on (**a**) germination rate, (**b**) plant height, (**c**) total fresh weight, and (**d**) disease class is shown. The concentration of the microbial antagonists was chosen according to the manufacturers´ instructions (Table 1), the products were mixed into the seed furrow. Healthy control: untreated seeds and soil; disease control: soil was inoculated with *R. solani* (0.5% and 1.5%) exclusively. All other treatments had soil inoculated with *R. solani* (0.5% and 1.5%) plus Trillus: *Pseudomonas fluorescens* (B177-M-03.08) & *Trichoderma harzianum* (B97-M-04.08), Vitalin Trichoderma: *T. harzianum* (T50), Serenade Aso: *Bacillus amyloliquefaciens* (QST713), T-Gro: *T. asperellum* (Kd), Vici Rhyzoteam: *Trichoderma* spp. n.s. + *Pseudomonas* spp. n.s. + *Glomus* spp. n.s., and Rhizo-Vital: *B. velezensis* (FZB42). The trial was done in three replicates with 30 seeds per treatment. The pots were incubated in the greenhouse at 20–25 °C and 50% − 65% relative humidity. Germination rate was assessed 21 days after sowing and the total fresh weight of the above-ground parts was assessed 35 days after sowing. Disease symptoms of *R. solani* on soybean hypocotyl were rated on a scale from class 0 to 4: 0 = no symptoms, 1 = scattered brownish discolouration, 2 = several slightly brownish spots or lesions, 3 = numerous distinct brown or black lesions, 4 = strong or extensive black lesions (Fig. 9). Different letters above the error bars indicate significant differences as per Tukey´s HSD test (*p* < 0.05) *n* = 90.

After establishing a bioassay for *R. solani*, the six most growth-suppressing microbial products identified in the in vitro experiments were mixed into *R. solani*-infected soil (Table 4). The soybean germination rate (Figs. 7 and 8a) decreased significantly from 93.3% in the healthy control plants to 57.5% in pots inoculated with 0.5% *R. solani* and to 45.2% in pots inoculated with 1.5% *R. solani*. None of the treatment significantly improved the impaired germination. Still, the addition of *T. harzianum* T50 (Vitalin Trichoderma) into the seed furrow improved germination rate by 13.6% for 0.5% *R. solani*-treated soil and treatment with *T. asperellum* (Kd; T-Gro), increased the germination rate of 1.5% *R. solani*-treated soil by 17.0% compared to the plants treated solely with 1.5%. *R. solani*. However, this was not significant. Plant height of healthy plants was highest and differed significantly from that of plants infected with both concentrations of *R. solani* (Fig. 8b). Antagonist treatment did not significantly alter plant height reduction caused by *R. solani*. Adding *T. harzianum (*T50) (Vitalin Trichoderma) or *T. asperellum* (Kd.; T-Gro) to the seed furrow tend to increased plant height. Regarding fresh weight, solely the addition of 1.5% *R. solani* led to a significant decrease in fresh weight, which was slightly but not significantly improved by the treatments *P. fluorescens* (B177-M-03.08) & T. *harzianum* (B97-M-04.08) (Trillus), *T. harzianum* T50 (Vitalin Trichoderma), *B. amyloliquefaciens (*QST713) (Serenade Aso), *T. asperellum* Kd (T-Gro), *T*. spp. + *P*. spp. + *Glomus* spp. (Vici Rhyzoteam) and *B. velezensis (*FZB42) (Rhizo Vital).

None of the plants in the healthy control displayed disease symptoms on the hypocotyl (Figs. 7 and 8d). In contrast, the plants in the pots inoculated with 0.5% *R. solani* displayed disease symptoms ranging from symptom class 1 to class 4. Only 8.9% of the plants were symptom-free after inoculation with *R. solani* (0.5%) and 2.7% after inoculation with 1.5% *R. solani* (Fig. 8d; Table 4). All microbial treatments increased the percentage of symptomless plants, this increase was significant for the treatment with *P. fluorescens* (B177-M-03.08) & *T. harzianum* (B97-M-04.08) (Trillus) at 0.5% *R. solani* and for *T. harzianum* (T50) (Vitalin Trichoderma) at 1.5% *R. solani*. The treatments increased the proportion of symptomless plants to 27,6% and to 21.1%, respectively. Additionally, heavy infections were reduced both treatments. Only 10.3% of the plants inoculated with *P. fluorescens* (B177-M-03.08) & *T. harzianum* (B97-M-04.08) (Trillus) displayed heavy infections compared to 28.9% in the 0.5% *R. solani* disease control. Inoculation with *T. harzianum* T50 (Vitalin Trichoderma) reduced heavy infections to 33.3%, compared to 51.4% in pots inoculated with 1.5% *R. solani*.

**Table 4:** Effect of microbial biocontrol products on percentage symptomless soybean plants cv. `Lenka´ infected with *Rhizoctonia solani* (AG-2-2-IIIB) (0.5%, 1.5%). See Fig. 8 for experimental conditions. Healthy control: untreated seeds and soil; disease control: soil was inoculated with *R. solani* exclusively. *B. am*.: *Bacillus amyloliquefaciens* (QST713); *B. ve*.: *Bacillus velezensis* (FZB42); *P. fl.*.: *Pseudomonas fluorescens* (B177-M-03.08); *T. asp*.: *Trichoderma asperellum* (Kd); *T. har*. (T50): *Trichoderma harzianum* (T50); *T. spp.*: *Trichoderma* spp. n.s.; control: treatment without antagonist. Different letters above the numbers indicate significant differences as per Tukey´s HSD test (*p* < 0.05), *n* = 90.

Treatment	Organism	*R*. solani 0.5%	*R*. solani 1.5%
Healthy control	**-**	100 ± 0.0^a^	100 ± 0.0^a^
Disease control	**-**	8.90 ± 9.3^c^	2.70 ± 6.57^c^
Trillus	*P. fl.*,* T. ha*.	**27.58 ± 8.9** ^**b**^	16.66 ± 21.60^bc^
Vitalin Trichoderma	*T. har.* (T50)	17.07 ± 16.65^bc^	**21.21 ± 0.93** ^**b**^
Serenade Aso	*B. am.*	10.52 ± 25.36^bc^	10.53 ± 10.00^bc^
T-Gro	*T. asp.*	12.09 ± 8.79^bc^	8.10 ± 15.35^bc^
Vici Rhyzoteam GR	*P.* spp., *Gl. spp.*,* T.spp.*	20.00 ± 16.77^bc^	22.2 ± 20.04^bc^
Rhizo Vital	*B.ve.*	15.79 ± 31.1^bc^	8.69 ± 7.71^c^

## Discussion

In this study, we evaluated the potential of registered microbial soil additives and commercial microbial plant protection products as antagonists against the soil-borne pathogens *R. solani*, *F. oxysporum*, *S. sclerotiorum* and *D. longicolla*, which have a negative impact on soybean production and contribute to the long-term deterioration of soil health^26–28^.

Bacterial and fungal strains were isolated from the microbial products to evaluate their antifungal potential in vitro. Here, *T.* spp. showed the highest and most consistent growth inhibition against the four soilborne pathogens. This aligns with the well-documented ability of *Trichoderma* to act as a biological control agent through rapid colonization, antibiosis, mycoparasitism, and the production of both antifungal enzymes and secondary metabolites^29–32^. The high inhibition rates observed here against all four pathogens (≥ 59%) confirm other findings reported for *Trichoderma* in dual culture assays against *Rhizoctonia*, *Fusarium*, and *Sclerotinia*^33–36^. The results where the inhibitory effects were lower (e.g. *T. harzianum & T. koningii* against *D. longicolla*) demonstrate the physiological diversity and strain specificity of the genus *Trichoderma*^37^. To our knowledge, only scarce peer-reviewed data are available about the biocontrol performance of the specific *Trichoderma* strains used in this study regarding the investigated pathogens and in soybean. Only for *T. harzianum* T58 antagonistic in vitro activity against *Phytophthora parasitica* has been shown^38^.

In contrast, the inhibitory effect of the bacterial strains on the mycelial growth of the four soilborne pathogens was lower and the strains displayed more heterogenous inhibition patterns. Only *B. amyloliquefaciens* and *B. velezensis* showed consistent inhibitory effects, in particular against *R. solani* and *D. longicolla*, reducing mycelial growth by at least 59%. The strong inhibition of *D. longicolla* (> 83%) is especially noteworthy, as only limited literature exists on antagonistic effects against this soybean-associated pathogen^39^. The genus *Bacillus* suppresses plant pathogens through various mechanisms such as the production of antibiotics, the synthesis of hydrolytic enzymes or the induction of systemic resistance^40–43^. All three tested *Bacillus* strains (*B. amyloliquefaciens*, *B. velezensis*, *B. atrophaeus*) exhibited chitinase, cellulase and protease activity, which directly contribute to cell wall degradation and mycoparasitism^44^. These enzymatic traits enhance direct antagonism in dual cultures where physical contact and enzymatic degradation plays an important role. Regarding the investigated strains a registration as fungicide agent against *R. solani* and *Sclerotinia* spp. is given in Germany for *B. amyloliquefaciens* QST713, but not for soybean production. Still, the general registration requires previous confirmation of its efficacy against both pathogens^45^. In general, QST713 is a well-described biocontrol agent, its activity against several plant pathogens has been described previously^46,47^. In contrast, the *Pseudomonas* strains displayed reduced (*P. fluorescens*) or absent enzymatic activity (*P. chlororaphis*). This finding is consistent with the common understanding that *Pseudomonas* mainly acts through secondary metabolites (e.g. phenazines, pyrrolnitrin, and hydrogen cyanide) rather than by extracellular enzyme activity^48^. This might explain why *Pseudomonas* strains showed weaker inhibition in dual culture compared to *Bacillus* and *Trichoderma* spp. As *P. chlororaphis* MA342 is registered as a fungicide against *Fusarium* spp. in cereals, its general biocontrol activity against this pathogen must have been shown before, but to the best of our knowledge no peer-reviewed data are available that extend these results regarding other pathogens^45^. Our findings thus broaden the functional spectrum of these commercial bacterial formulations for emerging European soybean systems.

The mechanism of biocontrol activity of the bacterial strains was further characterized by volatile organic compound (VOC) assays. VOC evaluation of the fungal antagonists *Trichoderma* spp. was not possible using the standard double-plate chamber method due to the rapid mycelial overgrowth of the whole plate. Given that *Trichoderma* VOCs and their broad-spectrum antagonistic mechanisms are already widely documented in the literature^49^ our focus was directed at the less characterized volatile profiles of the commercial bacterial formulations instead of assessing other available methods, such as charcoal-containing traps, or staggered inoculation times where the pathogen is pre-grown. Bacterial VOCs are recognized as important substances for long-distance antagonism^50,51^. Our assays revealed that all tested bacterial strains produced and emitted VOCs with potent, pathogen-dependent antifungal activity. From a practical agronomic perspective, evaluating these volatile traits directly serves our objective, as gaseous inhibition represents a key mechanism for field-ready products where direct physical contact with the pathogen is delayed. The antifungal effects of VOCs are mainly attributed to cell wall and membrane disruption, leakage of intracellular contents, and the induction of oxidative stress^52^.

The high sensitivity of *S. sclerotiorum* to VOC exposure corroborates with previous findings that this pathogen is vulnerable to volatile antifungal compounds^52,53^. The strong inhibition of *D. longicolla* (> 57%) is particularly noteworthy, as to the best of our knowledge, no studies have so far reported VOC-associated antagonistic effects against this soybean-related pathogen. While the VOC-mediated mycelial inhibition of *F. oxysporum* was weaker exposure to *Bacillus* strains and *Pseudomonas fluorescens* volatiles significantly decreased conidial production. This is in line with previous studies showing that VOCs can inhibit mycelial, conidiophore and conidial development^52,54^, thereby reducing inoculum abundance, infection pressure and pathogen spread in the field. Additionally, these volatiles induced a phenotypic shift in the *F. oxysporum* mycelial color from light pink to light yellow or cream. To the best of our knowledge, such VOC-associated changes in the mycelial pigmentation of *F. oxysporum* have not been reported previously.

In parallel, VOC exposure was accompanied by medium alkalization. Bacterial VOCs can induce environmental pH shifts^55^ and *Bacillus* and *Pseudomonas* strains are known for their ability to produce ammonia and nitrogenous volatiles^56,57^. These recognized pH changes may have contributed to fungal inhibition, as several pathogens are sensitive to alkaline stress^27^. However, as we did not observe pathogen growth impairment for *S. sclerotiorum*, *R. solani* and *F. oxysporum* after artificially increasing the pH of PDA plates to 7.5 without VOC challenge, we proved that their inhibition was purely VOC-mediated. For *D. longicolla* the inhibition of mycelial growth in the VOC assays was at least partly VOC mediated, as it was significantly higher than in the control assays at pH 7.5.

When microbial antagonists are used in integrated soybean inoculation, their compatibility with rhizobia, such as the genus *Bradyrhizobium* spp., has to be verified. Rhizobacteria colonize soybean roots, lead to nodule formation and are essential for nitrogen fixation, which in turn influences the overall protein content and soybean yield^23,58^. Compatibility tests with *Bradyrhizobium japonicum* and soybean cultivar `Lenka´ in vitro and in vivo revealed that most microbial antagonists did not affect rhizobial growth, soybean seed germination, plant growth, or root nodule formation. Only *P. chlororaphis* significantly reduced the germination rate and the number of root nodules. Our results show that the majority of the tested microorganisms are suitable for a combined application strategy with *B. japonicum*.

Subsequently, a soil-based bioassay for defined disease symptom development and monitoring upon inoculation with *R. solani* was developed. Inoculating the soil with 0.5% and 1.5% mycelial suspension of *R. solani* led to reproducible dose-dependent effects on the soybean plants. This included significant reductions in the germination rate and plant height, as well as increases in dark lesion symptoms on the hypocotyl. These results align well with previous findings on dose-dependent pathogenicity of *R. solani* on soybean seedlings^26^. Interestingly, while plant height and disease symptom scores responded strongly to pathogen load, total fresh weight did not differ significantly among treatments. This may reflect the high variability in biomass production at early growth stages or indicate that fresh weight is a less sensitive early indicator of *R. solani* infection.

When the microbial antagonists were introduced into the seed furrow of *R. solani*-infested soil, the ability of the microbial antagonists to reduce disease symptoms, such as brown lesions and impairment in plant development differed between the strains. Co-inoculation with *T. harzianum* (T50, Vitalin Trichoderma) partially improved germination rates and seedling height, although these biometric parameters did not reach statistical significance. *T. asperellum* kd. (T-Gro) showed similar but less pronounced effects, particularly under higher pathogen pressure, suggesting that its antagonistic activity may be more sensitive to pathogen density. In contrast, disease symptom assessments revealed positive effects upon treatment with *T. harzianum* T50 (Vitalin Trichoderma) and the microbial mixtures of *T. harzianum + P. fluorescens* (Trillus). Both treatments significantly increased the proportion of symptom-free plants and reduced heavy infections on the hypocotyl even at a higher pathogen concentration of 1.5%. However, none of the treatments significantly restored plant performance to the level of the healthy control.

This discrepancy, in which strong in vitro antifungal activity is only partially reproduced under in vivo conditions, is a well-documented phenomenon in biological control research^59,60^. While dual-culture and volatile assays isolate specific modes of action under optimized, nutrient-rich conditions, the soil matrix introduces complex biotic and abiotic interactions^61^. The finding that the antagonists significantly suppressed lesion development but did not rescue plant biomass suggests that despite localized pathogen restriction on the hypocotyl, the high disease pressure still imposed physiological stress on the developing seedlings during early growth stages.

Several factors likely contributed to the attenuated efficacy observed in the greenhouse trial. First, the native soil microbial community poses intense competition for space and nutrients, which can severely hinder the colonization and survival of the introduced strains (Weller, 1988). Second, physical and chemical soil properties can adsorb or degrade microbial secondary metabolites and extracellular lytic enzymes before they reach effective concentrations in the rhizosphere. Furthermore, while bacterial VOCs demonstrated powerful inhibition in closed double-plate systems, the highly porous and open soil architecture likely leads to rapid diffusion and dissipation of these volatile compounds, reducing their localized impact. Moreover, the observed differences between in vitro antagonism and *in planta* efficacy might be caused by the high concentration of *R. solani* (0.5% and 1.5%) used in this study, which very likely exceeded typical field infection levels. In agricultural fields, *R. solani* usually occurs in irregularly distributed infestation clusters. Consequently, our experimental setup represented a worst-case scenario. While this approach was methodologically necessary to ensure reproducible disease development, maximize statistical power and robust treatment comparisons^62,63^, it may underestimate the performance and real biocontrol potential of the tested microbial antagonists under natural field infection conditions. As candidate selection had to be based on the in vitro data to keep the experimental workload manageable, it cannot be excluded that candidates with superior in planta performance were overlooked.

Another important consideration is the method and rate of inoculant application. All microbial products were applied according to the manufacturer’s instructions, which were not specifically developed for soybean. Suboptimal crop-formulation compatibility or inoculum density, may have limited the establishment and persistence of certain antagonists in the soybean rhizosphere. Optimization of application strategies, such as seed coating, combined seed-soil treatments, or adjusted application rates, may therefore substantially improve field performance. Our results demonstrate that in vitro screening is an indispensable tool to identify specific antagonistic mechanisms, such as enzyme production or volatile emission, but it cannot fully predict complex rhizosphere interactions. Therefore, future research should focus on optimization strategies, including advanced formulation technologies or adjusted application timing, to transfer the antifungal potential of the investigated microbial strains into consistent in vivo plant protection.

In summary, we have shown that bacterial and *Trichoderma* strains provide antagonistic in vitro efficacy against four soil-borne fungal pathogens relevant in soybean production. Furthermore, under controlled greenhouse conditions, some *Trichoderma* strains showed disease-suppressive effects against *R. solani* by significantly reducing hypocotyl lesion severity under high disease pressure. While these controlled in vivo trials demonstrate the biological potential of these market-ready formulations, their practical field applicability remains to be validated. Therefore, future research must focus on elucidating the mechanisms underlying antagonist performance under varying pathogen loads. In addition, multi-location field trials are necessary to optimize application parameters, evaluate long-term impacts on crop yield, and establish robust, field-validated guidelines for soybean farmers to successfully integrate the analysed microbial products into soybean disease management strategies.

## Materials and methods

### Plant material and growth conditions

The experiments were performed with soybean (*Glycine max*) cv. `Lenka´, an early-maturing cultivar (maturity group 00). The seeds were provided by the Landesbetrieb Landwirtschaft Hessen (LLH), Germany. They were sown in commercially available soil (Floradur^®^, Floragard, Germany), mixed with sand in a 3:2 ratio (grain size 0–2 mm; Baustoffwerke Löbnitz GmbH, Germany) in plastic pots (9 cm × 9 cm × 9.5 cm, Göttinger, Germany). Total weight of the substrate was 300 g per pot. The soil was pre-heated for 24 h at 60 °C to eliminate fungus gnat eggs. Before sowing, the seeds were treated with a preparation of rhizobacteria (*Bradyrhizobium japonicum*, strains SEMIA 5079 and SEMIA5080, RHIZOLIQ^®^ Top S, BayWa, Germany) in combination with PREMAX^®^ (BayWa, Germany) to ensure adhesion of the rhizobia to the seeds. For each treatment, 30 seeds were placed in a Petri dish together with 12 µL of RHIZOLIQ^®^ Top S and 4 µL of PREMAX^®^ and shaken for five minutes. The seeds were then dried under a fume hood for ten minutes. Six seeds were sown per pot at a depth of 3 cm. To prevent cross-contamination of microbial agents through drainage water, pots assigned to the same treatment were grouped in shared trays (38 cm x 24 cm x 5.5 cm). To minimize potential positional and microclimatic edge effects within the greenhouse chamber, the arrangement of the pots on the benches was fully randomized and rotated on a daily basis. The plants were grown in the greenhouse at 20–25 °C and 50% to 65% relative humidity. The lighting was automatically adjusted so that the plants received at least 5 klux of light per day. All plants were watered on demand, up to three times a week.

### Microorganisms and growth conditions

The culture of *Fusarium oxysporum* (F.oxy 5 A) used in this study was isolated from Striga plants by Nzioki et al.^64^ in Western Kenya. The pathogen *Rhizoctonia solani* (AG-2-2-IIIB) was taken from the culture collection of the Julius Kühn Institute, Institute for Biological Control, Germany. *Sclerotinia sclerotiorum* (isolate 65263, JKI von Radnik, M., PSA) and *Diaporthe longicolla* (isolate 70833) from the main culture collection of the Julius Kühn Institute.

*Trichoderma* spp. were isolated from microbial products that are commercially available and registered either as fungicides or as soil additives (Table 1). The strains were grown at 26 °C on potato dextrose agar (PDA). Stock cultures of all fungal strains were maintained on PDA plates at room temperature. Fresh cultures were established by transferring a plug of stock mycelium onto a fresh PDA plate and incubating it for three days at 26 °C.

Bacterial strains were isolated from commercially available products (Table 1) by plating product suspensions on tryptic soy agar (TSA) and cultured for 72 h at 26 °C. Single colonies were subsequently transferred to fresh media and maintained as pure cultures. Working cultures on TSA were cultured at 20 °C and transferred every two weeks on fresh TSA plates. Permanent stock cultures were maintained at −80 °C in 70% glycerol. For products containing both bacterial and fungal microorganisms, selective media supplemented with antibiotics or fungicides, respectively, were used to obtain pure cultures of the target organisms. For *Trichoderma* strains, genus identity was further confirmed by isolating the fungal DNA with a DNeasy Plant Mini kit (Qiagen, Hilden, Germany). PCR amplification was performed using the primers ITS1 and ITS4, with cycles of 95 °C for 5 s, 55 °C for 20 s, and 72 °C for 20 s, followed by sequencing of the ITS region and comparison of the results with the NCBI data bank. For bacterial strains, no additional molecular identification was performed, and strain designation followed the information provided by the product manufacturers. To ensure strain purity and verify taxonomic consistency, all isolates were routinely monitored throughout the experiments based on their distinct colony morphology (color, shape, margin, and texture) and cell traits, which consistently matched the characteristic phenotypes of the respective genera.

### Dual culture tests

Dual-culture confrontation tests were done to assess the suppressive effect of bacteria and fungi against the pathogens *R. solani*, *F. oxysporum*, S. *sclerotiorum* and *D. longicolla*. A mycelial plug (8 mm diameter) of each fungal pathogen was taken from the edge of an actively growing colony and placed in the center of a PDA plate. In the *Trichoderma* spp. experiments, two mycelial plugs of the respective antagonists were placed on opposite sides of the PDA plate at a distance of 4 cm from the fungal pathogen plug, which were placed two days ahead of the antagonists. Mycelial plugs of the fungal pathogens alone on PDA served as control.

For experiments with bacterial strains, cells were taken from 48 h old colonies on Petri dishes with tryptic soy agar (TSA) using an inoculation loop and transferred to 5 mL of CASO broth (Carl Roth, Germany). The cultures were incubated for 24 h at 25 °C and shaking at 180 rpm (Minitron, Inforts HAT, Switzerland). Three sterile filter paper discs (5 mm diameter) were placed around the mycelial plug at a distance of 4 cm. A droplet of 15 µL of bacterial overnight culture was pipetted onto each filter paper disc two days after the pathogen plug was plated. The overnight cultures were previously adjusted with TSA to an optical density (OD)_600_ of 0.4. In the negative control, 15 µL CASO broth was pipetted onto the filter paper disc instead. The plates were incubated at 20 °C. For both *Trichoderma* spp. and bacterial antagonists, all experiments were conducted in triplicate and repeated at least three times. Plates were evaluated when the mycelial growth of the pathogen on the control plates reached the edge of the plate. To determine the inhibitory effect, the distance from the center of the pathogens mycelial plug to the outer fungal border was measured in a straight line to the filter plate or to the antagonist mycelium plug. The inhibition rate (IR) of the bacterial and fungal strains on pathogen growth was expressed as percentage (%) and was calculated according to Win et al.^65^ using the following formula

$$\:IR\:\left(\%\right)=\frac{RC-RA}{RC}\cdot\:100$$

IR = Inhibition rate (%).

RC = radius of the pathogen on the control plate.

RA = radius of the pathogen on the plates with antagonists.

### Volatile antifungal bioassay

The effects of volatile organic compounds (VOCs) produced by the bacterial strains on the four pathogens (*R. solani*, *F. oxysporum*, *S. sclerotiorum*, *D. longicolla*) were investigated by using the double-plate chamber method^66,67^. While fungal strains were initially considered for this assay, they had to be excluded because the fast-growing *Trichoderma* strains rapidly overgrew the physical boundaries of the chambers in preliminary tests, precluding a strictly volatile-mediated evaluation. Five bacterial strains were used in this study (Table 1). Briefly, 50 µL of overnight bacterial culture in tryptic soy broth (TSB) (OD_600_ = 0.4), prepared as described above, was spread on the surface of a TSA plate (9 cm diameter) and incubated for 72 h at 26 °C in the dark. A mycelial agar disc (8 mm diameter) from the edge of an actively growing colony of the fungal pathogen (*R. solani*, *F. oxysporum*, *S. sclerotiorum*, *D. longicolla*) was taken and placed in the center of a PDA plate. The inoculated PDA plates were incubated for 24 h at 26 °C in the dark. To set up the double-chamber assay, the lids of both plates (TSA with bacteria, PDA with fungal pathogen) were removed. The Petri dishes containing the fungal mycelial plug were then placed inverted over the bacterial plate. The double-chamber was sealed immediately with Parafilm (Amcor, Switzerland) to prevent leakage of volatile compounds. The average distance between the surfaces of the two plates was 2.0 cm. Control plates were set up in the same way, but without bacterial cultures on the TSA plates. All inoculated double-chambers were incubated at 26 °C in the dark for 72 h. The percentage inhibition of fungal growth by VOCs was calculated by measuring the mycelial diameter and using the formula according to^65^. The assays were performed three times, each with three replicates. In addition, the pH value of the PDA plates (containing the fungal pathogen) from the double-chamber was measured after 72 h using pH indicator sticks (Macherey- Nagel, Germany) by placing the sticks onto the surface of the medium. The number of conidia on the double-chamber plates with *F. oxysporum* was determined by flooding each PDA Plate with 1 mL of demineralized water. The spores were then detached from the mycelium using a scraper. The spore concentration was determined in a Fuchs-Rosenthal counting chamber (Carl Roth, Germany). This was done for all three plates, with three replicates each.

### Analysis of bacterial enzyme activity

All bacterial strains were screened for cellulase, chitinase and protease activity. While fungal antagonists were originally considered for these assays, they had to be excluded from the plate-screenings because the fast-growing *Trichoderma* strains rapidly overgrew the agar surface, preventing the reliable and precise measurement of localized clearing zones. For the cellulase assay the bacteria were plated on Carboxymethylcellulose agar (CMCA) containing KH2PO4 (1 g·L^− 1^), MgSO4 * 7H2O (0.5 g·L^− 1^), NaCl (0.5 g·L^− 1^); FeSO4* 7H2O (0.01 g·L^− 1^), MnSO4*H2O (0.01 g·L^− 1^), NH4NO3 (0.3 g·L^− 1^), Carboxymethylcellulose (10 g·L^− 1^), and agar: 12 g·L^− 1^. The pH was adjusted to 7.0 ± 0.2 prior to sterilization. For the protease assay, skimmed milk agar (SMA) plates were used containing peptone from casein (5.0 g·L^− 1^), yeast extract (2.0 g·L^− 1^), skim milk powder (28.0 g·L^− 1^), dextrose (1.0 g·L^− 1^), and agar (15.0 g·L^− 1^). The pH was adjusted to 7.0 ± 0.2. The composition of the medium for the chitinase assay was as follows: NaNO3 (2.0 g·L^− 1^), K2HPO4 (1.0 g·L^− 1^), MgSO4 (0.5 g·L^− 1^), KCl (0.5 g·L^− 1^), peptone from casein (0.2 g·L^− 1^), agar (15.0 g·L^− 1^), and chitin from crab shells (10 g·L^− 1^). Three sterile filter paper discs (6 mm diameter) were evenly distributed on each agar plate at a distance of 2.0 cm from the edge of the plate. Briefly, 15 µL of overnight bacterial culture in TSB broth (OD_600_ = 0.4), prepared as described above, was pipetted onto each filter paper disc. In the control plates without bacterial cultures, TSB served as control. The inoculated plates were incubated for 72 h at 26 °C in the dark. After the incubation period, the CMC and chitinase plates were flooded with Gram´s iodine solution (Carl Roth, Germany) for 15 min to visualize enzymatically degraded substrate. Transparent hydrolytic zones were observed against a red or brown background after Gram’s iodine staining respectively. Evaluation of protease activity was performed by visual assessment of the SMA plates for the formation of clear degradation zones around the filter discs. The enzyme activity was based on the presence (+) or absence (-) of a clearing zone. All assays were done in triplicate with three repetitions each.

### Analysis of antagonist compatibility with *Bradyrhizobium japonicum* and soybean plants

The compatibility of each of the microbial strain with *B. japonicum* and soybean seeds cv. `Lenka´ was checked in vivo. Therefore, 30 plants for each treatment (6 plants x 5 pots) were sown under the conditions as described above (see `Plant material and growth conditions´). The most effective microbial strains/products from the in vitro trials (Table 1) were poured into the seed furrow. To do so, the microbial preparations were dissolved in 200 mL water, and 30 mL of the resulting solution was then poured into the seed furrow of each pot. The microbial preparations were dosed and applied according to the manufacturer’s instructions (Table 1). In pots without added antagonists, 30 mL of water was poured into the seed furrow. After adding the microorganisms, the substrate surface was moistened on the surface with water from a spray bottle and then covered with sand. To prevent cross-contamination of microbial agents through drainage water, pots assigned to the same treatment were grouped in shared trays (38 cm x 24 cm x 5.5 cm). The pots were placed in a climate chamber at 10 °C in the dark until the first seeds germinated. Subsequently, the pots were incubated at 15 °C, with 50% to 65% relative humidity and 8.5 klux. To minimize potential positional and microclimatic edge effects within the climate chamber, the arrangement of the pots on the benches was fully randomized and rotated on a daily basis. The plants were watered on demand, up to three times a week. The germination rate and plant height were recorded 21 days after sowing. The plants were harvested after 35 days. The plants were carefully removed from the pots to keep the roots intact. The soil was then rinsed off under running water and the number of root nodules per plant was counted. The experiment was repeated three times.

### Development of a bioassay and seed inoculation with bacteria and fungi

**Fig. 9:**
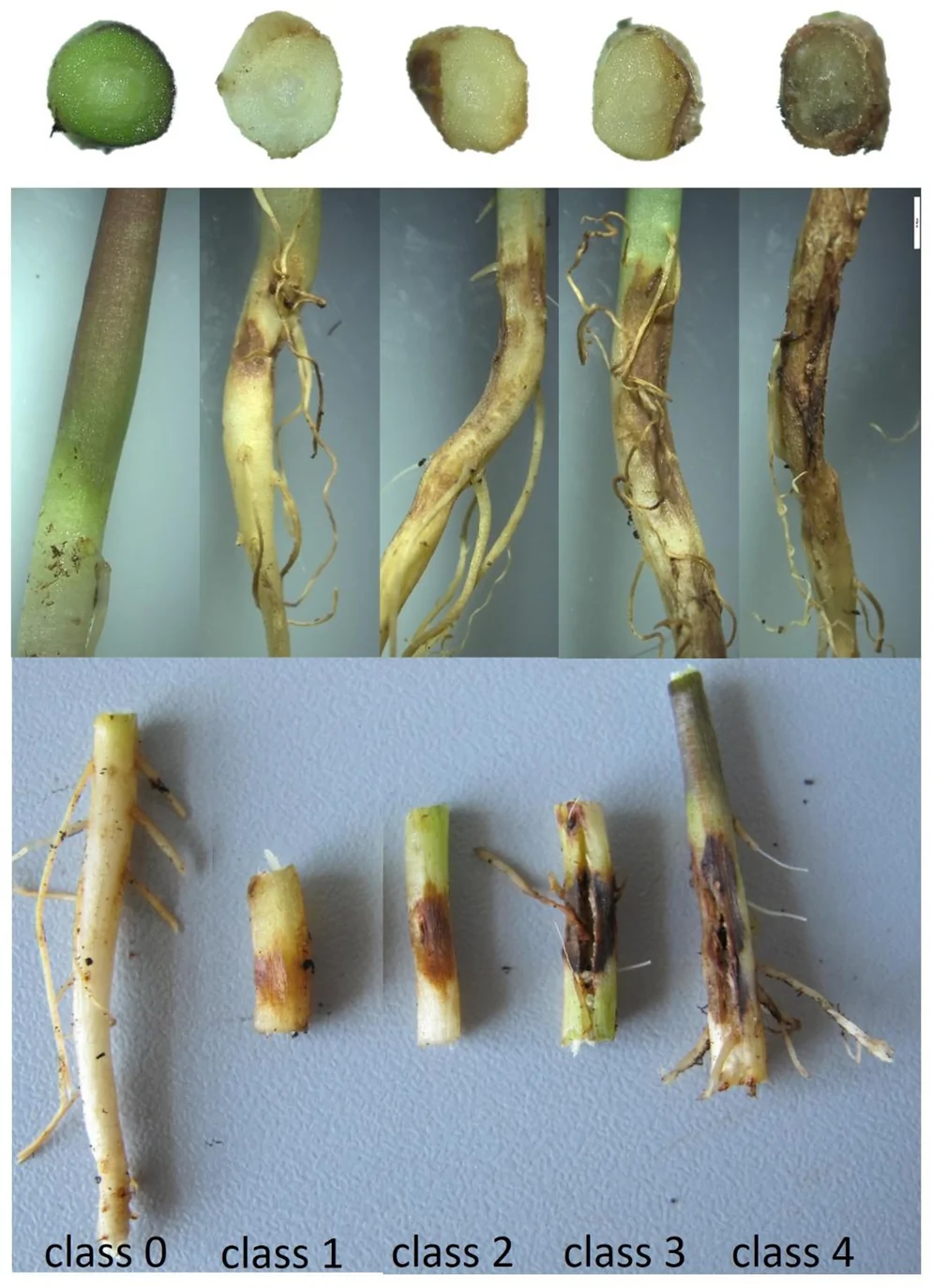
Rating scale of disease symptom classes on hypocotyl of soybean cv. `Lenka´ after 35 days of growth in soils inoculated with *R. solani*. 0 = no symptoms, 1 = scattered brownish discoloration, 2 = several slightly brownish spots or lesions, 3 = numerous dis-tinct brown or black lesions, 4 = strong or extensive black lesions.

In order to analyze disease-suppressive effects of bacterial and fungal strains in vivo, a pathosystem for *R. solani* was developed. The *R. solani* strain was used for the inoculation of soybean cv. `Lenka´ as follows: 200 mL of PDB was filled into a 1 L Erlenmeyer flask and autoclaved (20 min, 121 °C). The nutrient medium was then inoculated with an agar plug (8 mm diameter) from an actively growing *R. solani* culture and incubated for 7 days at 26 °C in the dark. The mycelium was collected by filtration through a funnel lined with cheesecloth and dried for 2 h. 1 g of mycelium was added to 10 mL of distilled water (10% suspension) and ground with a hand blender until the mycelium pieces were homogeneous in size. This 10% suspension was then mixed with the potting substrate at concentrations of 0.5%, 0.75%, and 1% suspension (mL·g^− 1^ soil). After inoculating the potting substrate with the pathogen, 30 plants for each treatment (5 pots) were sown under the conditions as described above (see “Plant material and growth conditions”). The most effective bacterial and fungal strains from the in vitro trials were poured into the seed furrow and the pots were incubated and watered as described before (see “Analysis of antagonist compatibility with *B. japonicum* and soybean plants”). The germination rate was recorded ten days after sowing. Plant height was measured after 10 days and 21 days of incubation. The plants were harvested after 35 days. The fresh weight of the above-ground parts was determined immediately after collection. The plants were then dried for three days at 60 °C in a drying cabinet, after which their dry weight was determined. The relative water content was calculated from the difference between the fresh and dry weights. Assessment of hypocotyl rot symptoms was carried out five weeks after inoculation. The infestation severity was estimated for each plant and categorized into disease symptom classes 0 to 4 (Fig. 9) using a modified scoring system established by Ludovic et al.^68^. The disease categories were defined according to the following scheme: 0 = no symptoms, 1 = scattered brownish discoloration, 2 = several slightly brownish spots or lesions, 3 = numerous distinct brown or black lesions, 4 = strong or extensive black lesions. All experiments were performed with three independent biological replicates, each consisting of five pots with six plants per pot for each treatment (30 plants). The experiment was repeated three times.

### Statistical analysis

All data were analyzed using the statistical software R (version 4.3.1) and the mean values were compared. First, the underlying assumptions for parametric methods were verified: normal distribution was assessed using the Shapiro-Wilk test, and homogeneity of variance was evaluated using Levene´s test. If both assumptions were met, data were subjected to a one-way ANOVA with `treatment´ as the independent variable followed by a post-hoc Tukey´s HSD test for pairwise comparisons. If the requirements for homogeneity of variance were violated, non-parametric methods were applied. In these cases, group comparisons were performed using the Kruskal-Wallis test followed by a post-hoc Dunn´s test with Bonferroni correction. For all tests, *p*-values below 0.05 were considered statistically significant. The compact letter display (CLD) for significant differences was generated based on the results of the post-hoc pairwise comparison tests.

## Supplementary Information

Below is the link to the electronic supplementary material.


Supplementary Material 1


## Data Availability

The datasets used and/or analyzed during the current study are available from the corresponding author upon reasonable request.
