# Supplementary material for: Trichoderma spp., Bacillus spp. and Pseudomonas spp. isolated from commercial products show biocontrol activity against soil-borne pathogens in soybean
Source: Sci Rep. 2026 Aug 28;16:27089. doi: 10.1038/s41598-026-67813-2 (PMC13524920; doi:10.1038/s41598-026-67813-2)
Supplement: Supplementary file 1 [file 41598_2026_67813_MOESM1_ESM.docx]

**Supplementary data**

***Trichoderma* spp*.*, *Bacillus* spp. and P*seudomonas* spp. isolated from commercial products show biocontrol activity against soil-borne pathogens in soybean**

Franziska Porsche^1^, Marwin S. Nagel^1^, Anja Frank^1^, Andreas Hammelehle^2^, Ada Linkies ^1,^*

^1^Julius Kühn-Institut (JKI), Bundesforschungsinstitut für Kulturpflanzen, Institut für Biologischen Pflanzenschutz, Schwabenheimer Str. 101, 69221 Dossenheim; franziska.porsche@julius-kuehn.de; anja.frank@julius-kuehn.de; marwin.nagel@gmx.de

^2^ Landesbetrieb Landwirtschaft Hessen, Fachinformation Ökologischer Landbau, Beratungsstelle Friedberg, Homburger Straße 17, 61169 Friedberg; andreas.hammelehle@llh.hessen.de

*****Correspondence: ada.linkies@julius-kuehn.de

**Table S1.** Mean pH values measured in bacterial volatile organic compound (VOC) assays. The pathogens *Rhizoctonia solani* (AG-2-2-IIIB), *Fusarium oxysporum* (F. oxy 5A), *Sclerotinia sclerotiorum* (65263 JKI), and *Diaporthe longicolla* (70833) were applied simultaneously with bacterial antagonists on opposite plates of Petri dishes and sealed with parafilm. pH values were determined after three days incubation at 26 °C. n = 9.

|  | Measured pH | | | |
| --- | --- | --- | --- | --- |
| Organism | *R. solani* | *F. oxysporum* | *S. sclerotiorum* | *D. longicolla* |
| Untreated control | 5.5 | 5.6 | 5.5 | 5.75 |
| *P. fluorescens* (B177-M-03.08 | 7.5 | 7.7 | 7.5 | 7.5 |
| *P. chlororaphis* (MA342) | 7.5 | 7.5 | 7.5 | 7.75 |
| *B. amyloliquefaciens* (QST713) | 7.5 | 7.4 | 7.5 | 7.5 |
| *B. velezensis* (FZB42) | 7.5 | 7.6 | 7.5 | 7.75 |
| *B. atrophaeus* n.s. | 7.5 | 7.5 | 7.5 | 7.75 |

**Figure S1.** Effect of bacterial and fungal strains on growth parameters of soybean plants cultivar `Lenka´. The microbial preparations were dosed and applied according to the manufacturer's instructions. Two replicates were performed, each with 30 plants per treatment. The pots were incubated at 10 °C in the dark until the first seeds germinated; subsequently, the pots were incubated at 15 °C under 8.5 klux. Germination rate (a) and plant height (b) were assessed 21 days after sowing, root nodules (c) were counted 35 days after sowing. The lletters next to the barsindicate significant differences (ANOVA for germination rate and plant height, post-hoc Dunn´s test for root nodules/plant*, p* < 0.05), n = 60.
